# Comparative study of mandibular linear measurements obtained by cone beam computed tomography and digital calipers

**DOI:** 10.4317/jced.51426

**Published:** 2014-07-01

**Authors:** Pablo Tarazona-Álvarez, Javier Romero-Millán, David Peñarrocha-Oltra, María Á. Fuster-Torres, Beatriz Tarazona, Miguel Peñarrocha-Diago

**Affiliations:** 1 Master in Oral Surgery and Implantology. Valencia University Medical and Dental School. Valencia, Spain; 2 Master in Oral Surgery and Implantology. Professor of the Master in Oral Surgery and Implantology. Valencia University Medical and Dental School. Valencia, Spain; 3 Associate Professor of Orthodontics. Valencia University Medical and Dental School. Valencia, Spain; 4 Chairman of Oral Surgery. Director of the Master in Oral Surgery and Implantology. Valencia University Medical and Dental School. Valencia, Spain

## Abstract

Objectives: Cone beam computed tomography (CBCT) is an innovative dental of imaging system characterized by rapid volumetric imaging with patient exposure to a single dose of radiation. The present study was carried out to compare the linear measurements obtained with CBCT and digital caliper in 20 mandibles from human cadavers. Study design: A total of 4800 linear measurements were measured between different mandibular anatomical points with CBCT and digital caliper. The real measurements were defined as those obtained with the digital caliper. Posteriorly, the mandibles were scanned to obtain the CBCT images, with software-based measurements of the distances. Results: The measurements obtained with the digital caliper were greater. The CBCT technique underestimated distances greater than 100 mm. Conclusions: CBCT allows to obtain linear mandibular anatomical measurements equivalent to those obtained with digital caliper. The differences existing between both methods were clinically acceptable.

** Key words:**Computed tomography, cone beam CT, accuracy, reliability, digital caliper.

## Introduction

Cone beam computed tomography [CBCT] is an image scanning and volumetric reconstruction technique that allows us to obtain linear measurements in three dimensions using computer software (1). CBCT can be used in different fields in dentistry, such as implantology and orthodontics (2). However, in order to optimize application of the technique, it is necessary to analyze the accuracy of the data obtained on performing linear measurements (3). The literature contains several studies comparing the linear distances recorded on the surface of models with those obtained by CBCT. They found statistically significant but clinically irrelevant differences (4), with errors ranging from 0.01-0.85 mm (5,6). However, few studies have compared the measurements obtained by CBCT imaging versus the real or actual measurements recorded from mandibles in cadavers (5,7).

The present study compares the linear measurements obtained by CBCT and digital caliper in 20 mandibles from human cadavers.

## Material and Methods

**Figure 1:**
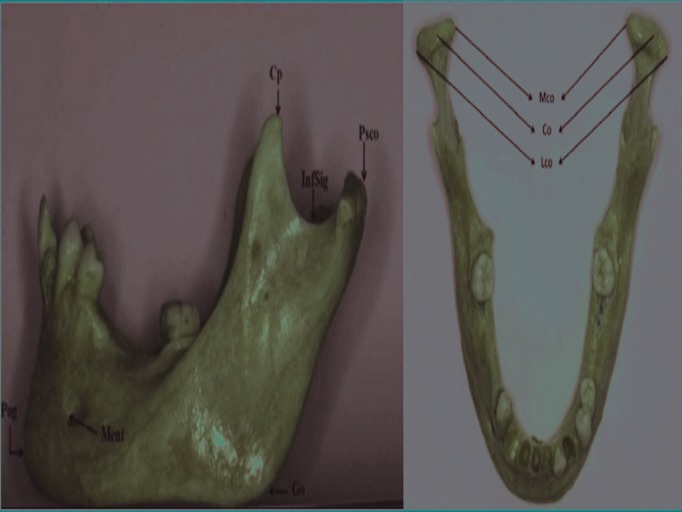
Anatomical points used as references for the measurements. Sagittal and coronal views.

20 mandibles from human cadavers supplied by the Department of Anatomy [Valencia University Medical and Dental School, Valencia, Spain] were used to obtain 20 linear measurements from different anatomical points described in  Table 1. Figure 1 shows the different anatomical landmarks in the sagittal and coronal views.

**Table 1:**
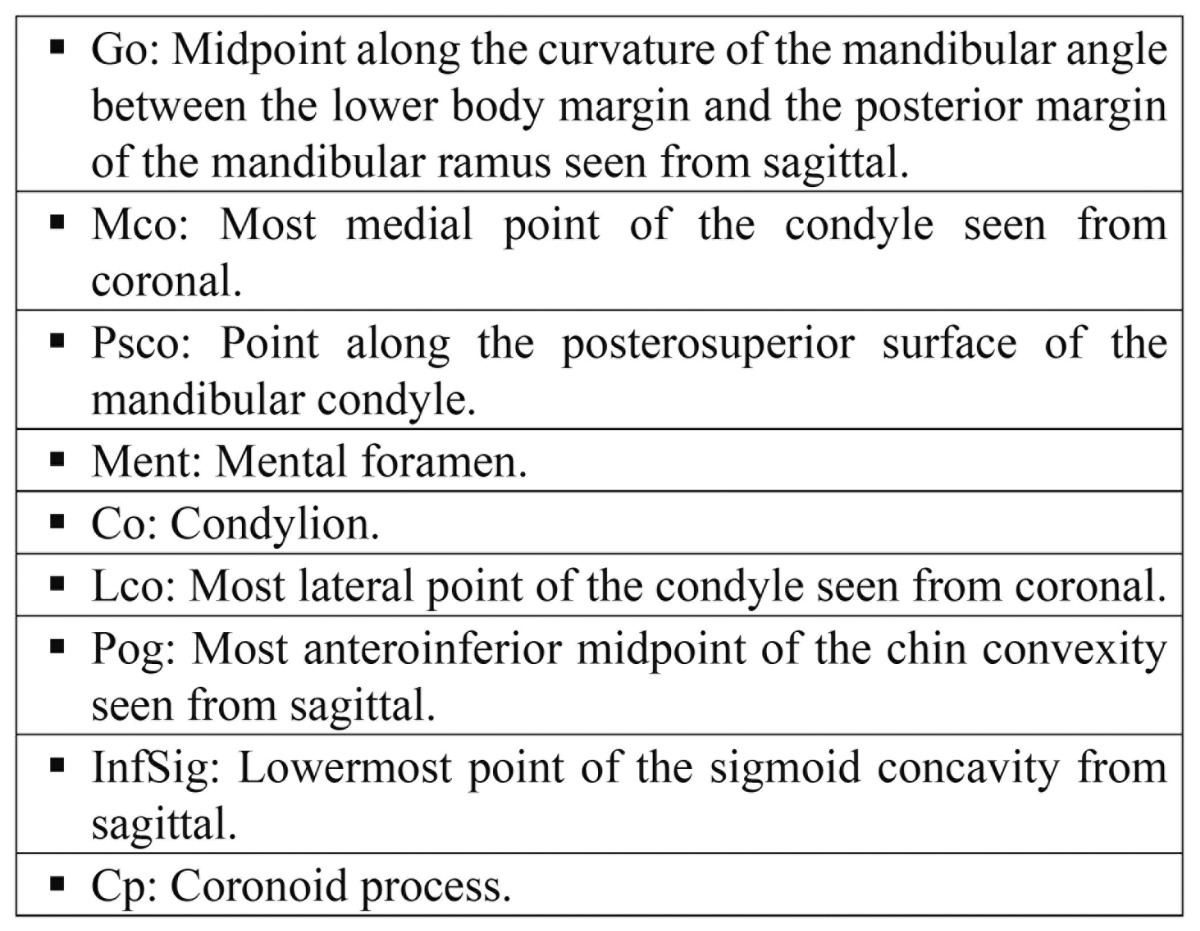
Anatomical points used as references for the measurements.

**Figure 2:**
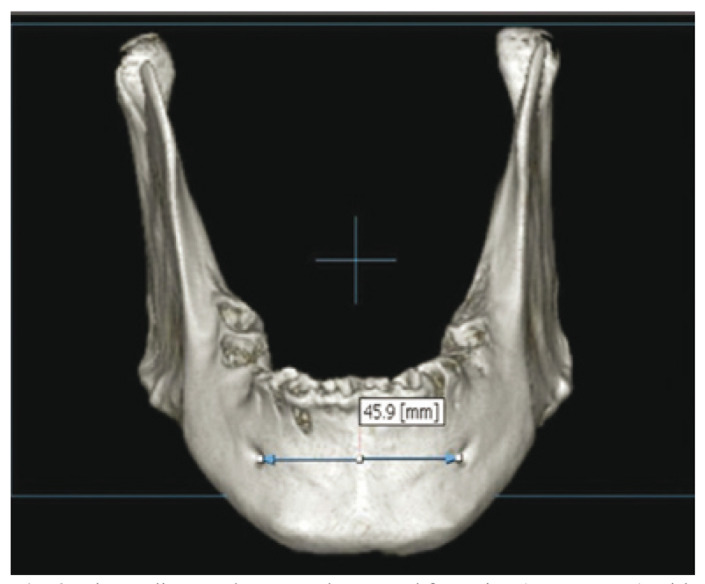
Linear distance between the mental foramina (Ment-Ment) with CBCT.

The measurements on the CBCT images were the same as those carried out on the mandibles with the digital caliper. A high precision digital caliper with a standard error of 0.02 mm [Halfords Advanced Professional Vernier®, United Kingdom] was used to obtain the real measurements from the human mandibles. Each mandible was scanned with the Picasso Dental Master 3D® system [Ewoo technology, Republic of Korea, 2005]. Imaging was performed with a voxel resolution of 0.1 mm and a field of view [FOV] of 20 x 19 cm, with 80 kVp and 5 mA irradiation, and an exposure time of 12-24 seconds. The images obtained were 1 mm axial or sagittal acquisitions and coronal reconstructions. The software was Ezimplant® [Vatech, Yongin-Si, Republic of Korea] (Fig. 2).

All mandibles were examined independently by two observers. Each observer measured the distances in triplicate using both Ezimplant® software [Vatech, Yongin-Si, Republic of Korea] and digital caliper, with calculation of the averages of the distances obtained by the two observers with both methods.

- Statistical analysis

The intraclass correlation coefficient was used to determine interobserver error. The t-test with Bonferroni correction was performed for analysis and comparison of the 20 measurements made on the 20 mandibles by the two observers. The level of significance was p ≤ 0.0025. All calculations were performed using the Statistical Package for Social Sciences [SPSS version 15, Chicago, IL, USA].

## Results

The linear measurements obtained in the mandibles with both the digital caliper and CBCT are described in  Table 2. The intraclass correlation coefficient ranged from 0.941-0.998 mm in the measures analyzed using CBCT and from 0.974-0.999 mm in the measures obtained with the digital caliper, reflecting high reliability between the two observers.

**Table 2:**
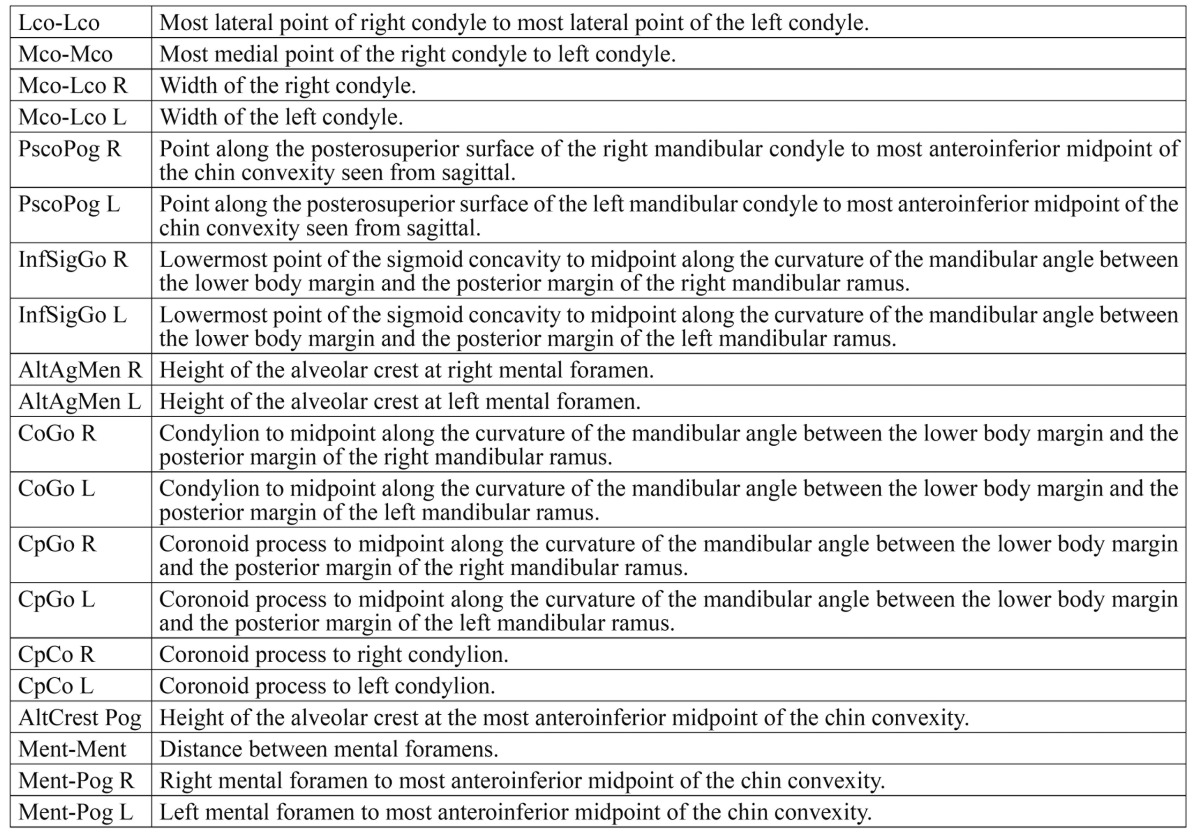
Description of the measures used between different anatomical points.

Comparison of the linear measurements made with digital caliper and CBCT showed no significant differences for 95% of the distances analyzed [*p*>0.0025], though differences were recorded for the remaining 5% [*p*<0.0025]. The average discrepancy between the linear distances obtained with both methods ranged from 0.001-0.82 mm. Observer 1 did not report statistically significant differences for any of the 20 distances analyzed. However, CBCT underestimated the actual measurements obtained with the digital caliper in 17 of the 20 distances analyzed ( Table 3). Observer 2 in turn reported a statistically significant discrepancy for one of the 20 linear distances [PscoPogL, *p*=0.000] ( Table 3), and in the same way as the Observer 1, CBCT underestimated the actual measurements obtained with the digital caliper in 16 of the 20 distances analyzed ( Table 3). Only one distance [PscoPogL] had a statistically significant discrepancy between the two methods ( Table 3).

**Table 3:**
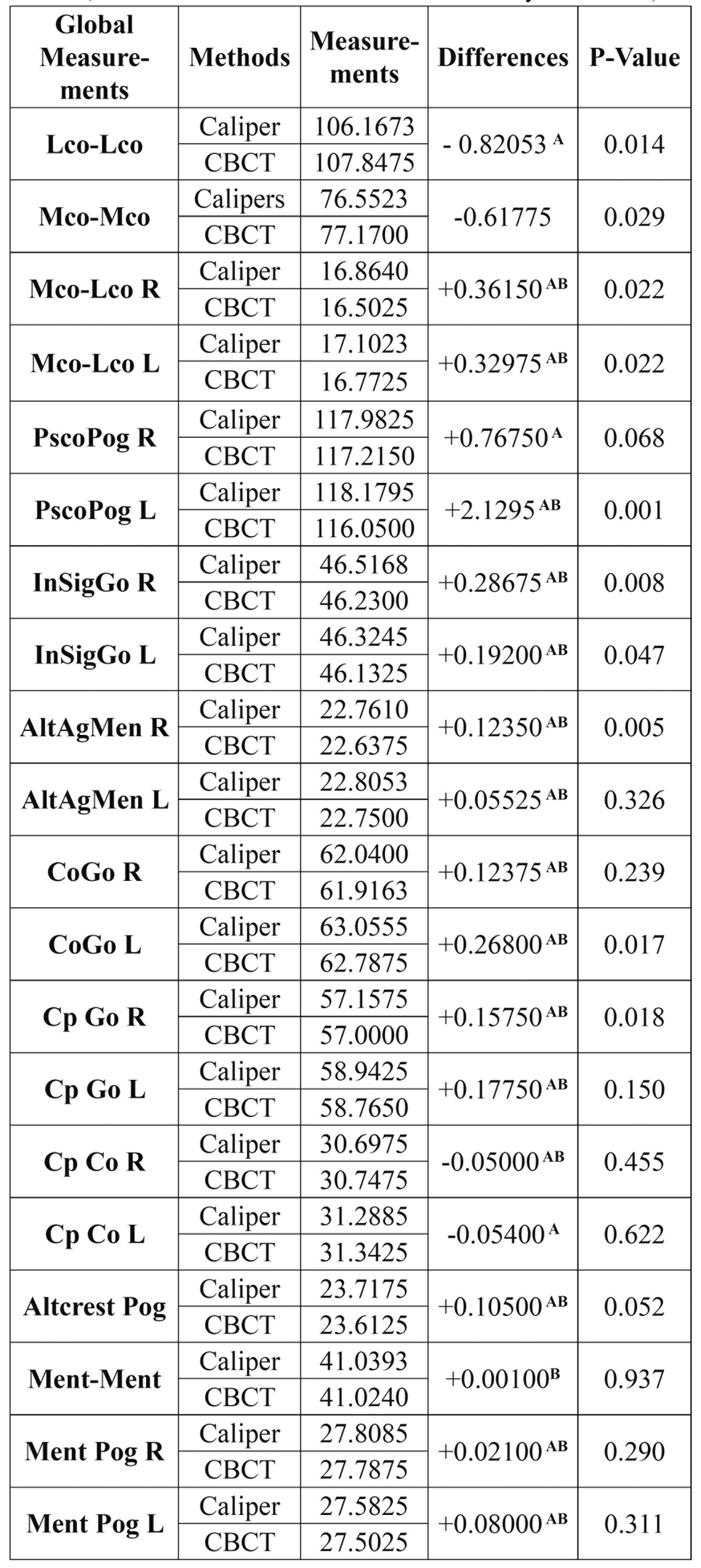
Linear measurements obtained in the mandibles with digital caliper and CBCT (X A: CBCT underestimated measurements by Observer 1; X B: CBCT underestimated measurements by Observer 2).

## Discussion

Mandibular linear measurements has been analyzed previously with CBCT and compared with two-dimensional techniques (5). However few studies have analyzed the accuracy of CBCT in human skulls. In the present study the two observers made 4800 linear measurements, and only 240 [5%] showed significant differences between analysis performed with the digital caliper and the measurements obtained by CBCT. The remaining 4560 measurements [95%] showed no statistically significant differences between the two techniques, the observed minimum discrepancy [5%] being considered clinically acceptable (8). Hilgers *et al*. (9) reported good accuracy and reliability of CBCT in a study of 11 measurements in 25 mandibles of cadavers, though there were significant discrepancies in some of their measurements. In the same line, Moreira *et al*. (7) found no statistically significant differences between CBCT and digital caliper in their measurements of 15 human skulls, in coincidence with the findings published by Kamburoglu *et al*. (10).

In our study CBCT was seen to underestimate the true measurements, though the difference was only statistically significant for distances greater than 100 mm and in low density zones such as the condyle. Distances with important but nonsignificant differences may be due to several causes, e.g., areas of low density secondary to dehydration that are not exactly reproducible by CBCT and therefore cannot be accurately analyzed by the Ezimplant® software (11), or measurements in excess of 100 mm (12).

In 60% of the measurements made with two types of CBCT, Damstra *et al*. (4) observed a tendency to underestimate with dry mandibles respect to the reference measurements. In the same line, two studies (13,14) with human adult skulls or synthetic mandibles also recorded a underestimation with CBCT images. Other authors (3,15,16), in obtaining measurements of structures inside the skull, also reported underestimation with the use of CBCT. However Baumgaertel *et al*. (17) reported systematic overestimation when analysis was performed on a segmented basis. A possible explanation for this may be the software used [Ezimplant®, Vatech, Yongin-Si, Republic of Korea]. Glover *et al*. (11) offered another possible explanation, the so-called partial volume effect, which occurs when a voxel is occupied by two structures with two different densities, and CBCT records an average of both for that voxel. Thus, when a voxel exhibits two different densities, the resulting end density is the average of both structures, with interpretation of part of one structure or the other.

## Conclusions

CBCT is a reliable and accurate method for analyzing linear measurements made on mandibles of human cadavers. It must be taken into account that there were statistically nonsignificant discrepancies in the linear measurements analysis. However, further studies are needed to determine the cause of the results founded in this study.

## References

[1] Cavalcanti MG, Rocha SS, Vannier MW (2004). Craniofacial measurements based on 3D-CT volume rendering: implications for clinical applications. DentomaxillofacRadiol.

[2] Zamora N, Cibrián R, Gandia JL, Paredes V (2013). Study between anb angle and Wits appraisal in cone beam computed tomography (CBCT). Med Oral Patol Oral Cir Bucal.

[3] Lascala CA, Panella J, Marques MM (2004). Analysis of the accuracy of linear measurements obtained by cone beam computed tomography (CBCT-NewTom). Dentomaxillofac Radiol.

[4] Damstra J, Fourie Z, Huddleston Slater JJ, Ren Y (2010). Accuracy of linear measurements from cone-beam computed tomography-derived surface models of different voxel sizes. Am J Orthod Dentofacial Orthop.

[5] Ludlow JB, Laster WS, See M, Bailey LJ, Hershey HG (2007). Accuracy of measurements of mandibular anatomy in cone beam computed tomography images. Oral Surg Oral Med Oral Pathol Oral Radiol Endod.

[6] Kobayashi K, Shimoda S, Nakagawa Y, Yamamoto A (2004). Accuracy in measurement of distance using limited cone-beam computerized tomography. Int J Oral Maxillofac Implants.

[7] Moreira CR, Sales MA, Lopes PM, Cavalcanti MG (2009). Assessment of linear and angular measurements on three-dimensional cone-beam computed tomographic images. Oral Surg Oral Med Oral Pathol Oral Radiol Endod.

[8] Waitzman AA, Posnick JC, Armstrong DC, Pron GE (1992). Craniofacial skeletal measurements based on computed tomography: Part I. Accuracy and reproducibility. Cleft Palate Craniofac J.

[9] Hilgers ML, Scarfe WC, Scheetz JP, Farman AG (2005). Accuracy of linear temporomandibular joint measurements with cone beam computed tomography and digital cephalometric radiography. Am J Orthod Dentofacial Orthop.

[10] Kamburoğlu K, Kolsuz E, Kurt H, Kiliç C, Özen T, Paksoy CS (2011). Accuracy of CBCT measurements of a human skull. J Digit Imaging.

[11] Glover GH, Pelc NJ (1980). Nonlinear partial volume artefacts in x-ray computed tomography. Med Phys.

[12] Michael L (2005). Accuracy of linear temporomandibular joint measurements with cone beam computed tomography and digital cephalometric radiography. Am J Orthod DentofacialOrthop.

[13] Stratemann SA, Huang JC, Maki K, Miller AJ, Hatcher DC (2008). Comparison of cone beam computed tomography imaging with physical measures. Dentomaxillofac Radiol.

[14] Lagravère MO, Carey J, Toogood RW, Major PW (2008). Three-dimensional accuracy of measurements made with software on cone-beam computed tomography images. Am J Orthod Dentofacial Orthop.

[15] Brown AA, Scarfe WC, Scheetz JP, Silveira AM, Farman AG (2009). Linear accuracy of cone beam CT derived 3D images. Angle Orthod.

[16] Scarface WC, Farman AG, Sukovic P (2008). Clinical applications of cone-beam computed tomography imaging with physical measures. Dentomaxillofac Radiol.

[17] Baumgaertel S, Martin J, Palomo L, Hans MG (2009). Reliability and accuracy of cone-beam computed tomography dental measurements. Am J OrthodDentofacialOrthop.

